# Supplementary material for: Chromothripsis during telomere crisis is independent of NHEJ, and consistent with a replicative origin
Source: Genome Res. 2019 May;29(5):737–49. doi: 10.1101/gr.240705.118 (PMC6499312; doi:10.1101/gr.240705.118)
Supplement: Supplemental Material [file supp_gr.240705.118_Supplemental_file_1.zip › contigs/annotated_contigs/DB53/contig.2.DB53_length_443_mean_cov_13.7336343115.docx]

**DB53_length_443_mean_cov_13.7336343115**

```
CTTCGTTTCTTTTTGGTGCCTAGGGCACCATTTGACCAATGGCCTGGGGCCTCTTTGAATCTCTTCTCCTT|C|TGATGTGCAGGAAGC
 >chr8:75087895-75087967 - E=9e-32                                      >chr8:75095375-
CTCTGGGTGGGTGCTGGGCTCAAAGCTCCTCTTGCAATTCTTTTAGATTGGTCACCATCTTAATCATTTCCAGGTTATGCTGGTTCAGT
75095747 + E=9e-213
CAATGATCAGATATTTATTGAGCATGTACTTGAGGCAAGGCATCAAACTAGATATTGTAGAGAAACAAAAAGAAGTTTCAGTCACAGGC

TCTGCCCTTTAGATATTTGTAATCTCTTTGGGAGAGATGTTTCAGGTTGAAATAGGCATGGGAAATGTCAATTATATAAAACAACATTG

AGCATATAATATAATCATGAAGAACAGCCTTGCTACAAAATATTGGTAGTTCAGAGGATTAAATTAACACACACTAAGAGATGCCATGT
```
